# Hypercholesterolemia in Two Siblings with Resistance to Thyroid Hormones Due to Disease-Causing Variant in Thyroid Hormone Receptor (*THRB*) Gene

**DOI:** 10.3390/medicina56120699

**Published:** 2020-12-15

**Authors:** Maja Pajek, Magdalena Avbelj Stefanija, Katarina Trebusak Podkrajsek, Jasna Suput Omladic, Mojca Zerjav Tansek, Tadej Battelino, Urh Groselj

**Affiliations:** 1 Department of Paediatric Surgery and Intensive Care, University Medical Centre Ljubljana, Bohoričeva 20, 1000 Ljubljana, Slovenia; maja.jakic@gmail.com; 2 Department of Endocrinology, Diabetes and Metabolic Diseases, University Children’s Hospital, University Medical Centre Ljubljana, Bohoričeva 20, 1000 Ljubljana, Slovenia; magdalena.avbelj@mf.uni-lj.si (M.A.S.); katarina.trebusakpodkrajsek@mf.uni-lj.si (K.T.P.); jasna.suputomladic@kclj.si (J.S.O.); mojca.zerjav-tansek@mf.uni-lj.si (M.Z.T.); tadej.battelino@mf.uni-lj.si (T.B.); 3 Faculty of Medicine, University of Ljubljana, Vrazov trg 2, 1000 Ljubljana, Slovenia

**Keywords:** thyroid, resistance to thyroid hormones, RTHβ, thyroid hormone receptor, THRB, hypothyroidism, hypercholesterolemia

## Abstract

Resistance to thyroid hormone beta (RTHβ) is a syndrome characterized by a reduced response of target tissues to thyroid hormones. In 85% of cases, a pathogenic mutation in the thyroid hormone receptor beta (*THRB*) gene is found. The clinical picture of RTHβ is very diverse; the most common findings are goiter and tachycardia, but the patients might be clinically euthyroid. The laboratory findings are almost pathognomonic with elevated free thyroxin (fT4) levels and high or normal thyrotropin (TSH) levels; free triiodothyronin (fT3) levels may also be elevated. We present three siblings with *THRB* mutation (heterozygous disease-variant c.727C>T, p.Arg243Trp); two of them also had hypercholesterolemia, while all three had several other clinical characteristics of RTHβ. This is the first description of the known Slovenian cases with RTHβ due to the pathogenic mutation in the *THRB* gene. Hypercholesterolemia might be etiologically related with RTHβ, since the severity of hormonal resistance varies among different tissues and hypercholesterolemia in patients with *THRB* variants might indicate the relatively hypothyroid state of the liver. We suggest that cholesterol levels are measured in all RTHβ patients.

## 1. Introduction

Resistance to thyroid hormone beta (RTHβ) is a syndrome characterized by a reduced response of target tissues to thyroid hormones [1,2,3,4,5]. In 85% of cases, a pathogenic mutation in the thyroid hormone receptor beta (*THRB*) gene is found [3,4,5,6]. The remaining 15% meet the RTHβ criteria but the pathogenic mutation in the *THRB* gene is not found, suggesting that RTHβ might be due to mutations in gene encoding a regulatory cofactor, which has still not been proven [1,7,8].

The RTHβ incidence is approximately 1/40,000–50,000 births with familial occurrence in 75%, gender distribution is equal. The clinical picture of RTHβ is very diverse; the most common findings are goiter and tachycardia, but patients might be clinically euthyroid [1,2,6,9]. The laboratory findings are almost pathognomonic with elevated free thyroxin (fT4) levels and high or normal thyrotropin (TSH) levels; free triiodothyronin (fT3) levels may also be elevated [1,3,6].

It is known that hypothyroidism is associated with hypercholesterolemia with elevated low-density lipoprotein cholesterol (LDL-c) and total cholesterol (TC) levels, normal, elevated or reduced high-density lipoprotein cholesterol (HDL-c) levels and normal or elevated triglycerides and lipoprotein (a) (Lp(a)) levels and also non-alcoholic fatty liver disease [10].

We present two siblings with a pathogenic mutation in the *THRB* gene, who also had hypercholesterolemia, which can be etiologically related to RTHβ. This is also the first description of RTHβ due to the pathogenic mutation in the *THRB* gene in the Slovenian population.

## 2. Case Reports

A then 13.5 year-old girl was referred to our outpatient clinic due to suspicion of thyroid malfunction. Prior to that, she became ill with malaise, sub-febrile state (body temperature up to around 38.5 °C) and weight loss (3–4 kg in one month). Her body mass index (BMI) was 19.4 mg/kg^2^. She did not have diarrhea, palpitations, or a tremor. On physical examination, she was marginally tachycardic (96 beats/minute) and had a slightly enlarged thyroid gland. Measured thyroid hormone levels were: TSH 4.65 mIU/L (normally 0.55 to 4.78 mIU/L), fT4 39.9 pmol/L (11.5 to 22.7 pmol/L) and fT3 10.11 pmol/L (3.5 to 6.5 pmol/L); thyroid antibodies were absent. A slightly elevated TC level, which was 5.5 mmol/L (212.7 mg/dL) (normally less than 5 mmol/L (193.4 mg/dL)), was detected. LDL-c was borderline elevated (3.1 mmol/L (120 mg/dL)), while the HDL-c and triglycerides levels were in the normal range. The thyroid gland appeared enlarged with soft and elastic consistency and no nodules on palpation. The ultrasound showed an enlarged and thickened thyroid gland, isoechogenic, fine granular structure, without nodules and with normal perfusion. Family history was positive for thyroid disorders; hyperthyroidism was diagnosed in the girl’s mother and several relatives from the maternal side and they were all treated with thyrostatic drugs and/or radioactive iodine in past years. Targeted genetic testing was performed. We excluded pathogenic mutation in the thyroid-stimulating hormone receptor (TSHR) gene encoding TSH receptor and confirmed heterozygous pathogenic mutation in the THRB gene (NM_001128177.2: c.727 C>T; p.Arg243Trp), which is already described in the literature as pathogenic [11]. Consequently, we issued genetic testing in family members and the variant was confirmed in the girl’s mother, younger brother, and younger sister.

The younger sister was clinically euthyroid, on examination we detected marginal tachycardia (95 beats/min). Her BMI was 13.4 mg/kg^2^. Laboratory levels of thyroid hormones were TSH 10.46 mIU/L, fT4 35.81 pmol/L and fT3 14.16 pmol/L, thyroid antibodies were absent. The thyroid gland appeared to be an adequate size with soft consistency, smooth surface and without nodules on palpation. The thyroid ultrasound was unremarkable. She was referred to us a year later due to elevated cholesterol levels detected on preventive medical examination as part of a national screening. Elevated TC levels 6.6 mmol/L (254.8 mg/dL) and elevated LDL-c 4.2 mmol/L (162.2 mg/dL) (normally less than 3 mmol/L (115.8 mg/dL)) were detected. HDL-c and triglycerides were in a normal range. Familial hypercholesterolemia (FH) was not confirmed with genetic analysis (the low-density lipoprotein receptor (*LDLR*) gene, apolipoprotein B (*APOB*) gene, proprotein convertase subtilisin/kexin type 9 (*PCSK9*) gene).

The younger brother was also identified with this pathogenic mutation in the *THRB* gene as part of the family screening. His laboratory levels were: TSH 7.87 mIU/L, fT4 32.68 pmol/L and fT3 12.28 pmol/L, thyroid antibodies were absent. On clinical examination, marginal tachycardia was detected. The thyroid gland appeared slightly enlarged with elastic consistency and without nodules on palpation, which was confirmed with the ultrasound. Cholesterol levels were in a normal range. His BMI was 15.2 mg/kg^2^.

**Figure 1:**
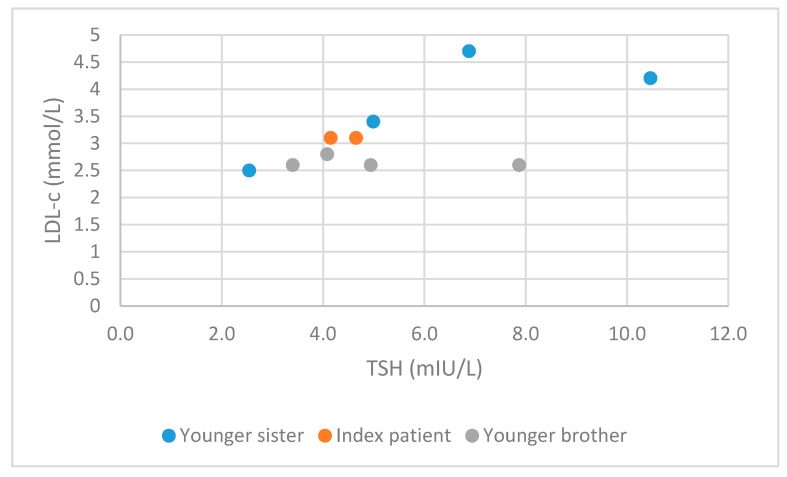
The correlation between all concurrent low-density lipoprotein cholesterol (LDL-c) and thyrotropin (TSH) measurements in our three siblings with confirmed thyroid hormone resistance due to pathogenic mutation in the thyroid hormone receptor beta (*THRB)* gene. Pearson correlation coefficient (*r*) was 0.62.

Table 1 represents a comprehensive summary of clinical and laboratory characteristics of the three subjects from all outpatient visits. The concurrent LDL-c and TSH levels measured in our subjects are presented in Figure 1. Pearson correlation coefficient was showing a large strength of association (*r* = 0.62).

In this family, there were no unaffected siblings. Unfortunately, there were no clinical or laboratory data available of the mother.

## 3. Discussion

RTHβ due to pathogenic mutation in the *THRB* gene is a rare congenital thyroid axis disease with predominant autosomal dominant inheritance pattern in 85% of patients [1]. To date, more than 170 different pathogenic mutations in the *THRB* gene have been described [5] with p.Arg338Trp as the most common one [12]. We describe a family with RTHβ due to p.Arg243Trp pathogenic mutation in the *THRB* gene previously reported as a cause of the RTHβ [11]. It is important to perform genetic tests in family members, with elevated levels of thyroid hormones, of a patient with whom pathogenic mutation in the *THRB* gene was confirmed [6]. Using genetic tests, we also certified RTHβ due to pathogenic mutation in the *THRB* gene in the mother (but no other clinical data were available), younger brother and younger sister.

It is known that thyroid disorders may increase the risk for cardiovascular diseases (CVD); hypothyroidism is associated with an increased cardiovascular risk, partially via impact on the individual’s lipids profile and hyperthyroidism via increased cardiac output, wider pulse pressure and tachycardia [10]. Tachycardia and palpitations are one of the most frequent clinical signs of RTHβ due to pathologic mutation in the *THRB* gene presenting in 33–75% of patients, with a characteristic euthyroidism in other tissues [2,3,6,9,13]. This can be explained with various isoforms of THR dominating in different tissues [6]. Thyroid hormone receptors (THR) are ligand depended transcription factors. *THRA* and *THRB* genes are known. Mutations in the *THRB* gene are the cause of RTHβ as mentioned; *TRHA* gene mutations have been recently discovered, causing major abnormalities in growth and gastrointestinal function [5,14]. Alternative splicing of primary transcripts leads to four T3 binding isoforms (β1, β2, β3 and α1), which are expressed in different tissues. For understanding this case, the most important isoforms are TRβ1, which is mostly expressed in the liver, kidneys and thyroid, and TRα1, which is mostly expressed in the heart, bones and brain [5,9,14]. The severity of hormonal resistance varies among different tissues. Tachycardia, as one of the most frequent clinical signs of RTHβ, can be explained with high endogenous levels of thyroid hormones leading to local thyrotoxic conditions particularly in tissues that predominantly express TRα1, such as the heart. Tachycardia was also present in all the siblings reported, but no treatment was necessary. In our index patient and her younger sister, hypercholesterolemia with elevated TC levels was detected. The younger sister also had elevated LDL-c levels. It is known that hypothyroidism is associated with hypercholesterolemia with elevated LDL-c and TC levels, normal, elevated, or reduced HDL-c levels and normal or elevated triglycerides and Lp(a) levels and also non-alcoholic fatty liver disease. Thyroid hormones have a direct effect on fatty acid β-oxidation, cholesterol synthesis, lipogenesis and the reverse cholesterol transport pathway. In vivo studies have also suggested that when TSH levels are high, TSH binds to TSHR in the liver and modulates hepatic lipid and cholesterol homeostasis. These in vivo studies showed direct action of TSH independently from thyroid hormones, which is not the case in living human beings, therefore the interpretation is difficult. All these effects are mediated by thyroid hormones at the transcriptional and post-translational level and also via autophagy. As previously mentioned, the predominant isoform in the liver is TRβ1 [5,9,11,15]. In patients with RTHβ, the liver is relatively resistant to thyroid hormones, which might indicate the relatively hypothyroid state of the liver [15]. According to our clinical data, there is a large strength of association between TSH and LDL-c levels. This all leads to the possible conclusion that thyroid hormone analogues may offer a therapeutic option for adjuvant hypercholesterolemia treatment in these patients [15]. Hypercholesterolemia with elevated LDL-c levels, slightly elevated triglycerides, and reduced HDL-c levels in patients with RTHβ was previously described by Mitchell, C.S. et al. [16]. Owen et al. also described elevated LDL-c levels and also increased arterial stiffness in patients with RTHβ compared to the euthyroid population [17].

There are also other possible causes for hypercholesterolemia independent from RTHβ that need to be considered. One important cause of hypercholesterolemia is FH, which was not confirmed with genetic analysis in the index case. According to our data, one in 500 children born in 2008 has genetically confirmed FH. There is about 50% probability of children with hypercholesterolemia having FH [18,19]. There are also other possible causes of hypercholesterolemia; one of the most important causes in the adult population is obesity [20]. Iqbal, A.M. et al. also described that severely obese children with higher TSH levels also have higher TC and HDL-c levels compared to those with normal TSH [21]. Other rare causes of hypercholesterolemia in children may be metabolic diseases (such as lysosome storage disorders), renal diseases (such as nephrotic syndrome), liver diseases, and the use of various medications (e.g., diuretics, glucocorticoids, immunosuppressives, and oral contraceptives) [21,22]. Our patients had no indications for any of the above-mentioned causes of hypercholesterolemia.

Recognition and appropriate RTHβ treatment can be challenging. Amor, A.J. et al. published that 19% of patients with RTHβ were mistreated [12]. Treatment with thyrostatic drugs may exacerbate RTHβ symptoms, inhibit the growth and increase goiter size and is thus not recommended [23]. As most of the other reported cases with RTHβ, our cases also did not require any treatment.

## 4. Conclusions

We presented three siblings with RTHβ due to pathogenic mutation in the *THRB* gene discovered for the first time in our population. Mild to moderate hypercholesterolemia was detected in two out of three screened family members with confirmed pathogenic mutation in the *THRB* gene, which might be etiologically related. Of note, the most common genetic etiology of FH was excluded in the index case. Since the severity of hormonal resistance varies among different tissues, one possible explanation could be that hypercholesterolemia in patients with THRB variants might indicate the relatively hypothyroid state of the liver. We suggest that cholesterol levels are measured in all RTHβ patients. Further studies are needed to appropriately address the CVD risk profile in RTHβ patients.

## Figures and Tables

**Table 1:** Laboratory and clinical characteristics of three siblings with confirmed thyroid hormone resistance due to pathogenic mutation in the thyroid hormone receptor β gene.

	Siblings with Confirmed *THRB* Mutation		
	Index Patient	Younger Sister	Younger Brother
Clinical signs and symptoms	Malaise, subfebrile state, weight loss, marginal tachycardia	Asymptomatic, marginal tachycardia	Asymptomatic, marginal tachycardia
Peak thyroid hormone levels	TSH 4.65 mIU/L (B)	TSH 10.46 mIU/L (E)	TSH 7,87 mIU/L (E)
	fT4 39.9 pmol/L (E)	fT4 35.81 pmol/L (E)	fT4 32.68 pmol/L (E)
	fT3 10.11 pmol/L (E)	fT3 14.16 pmol/L (E)	fT3 12.28 pmol/L (E)
Thyroid antibodies	Absent	Absent	Absent
Peak cholesterol levels	TC 5.5 mmol/L (E)	TC 6.6 mmol/L (E)	TC 4.5 mmol/L (N)
	LDL-c 3.1. mmol/L (B)	LDL-c 4.2 mmol/L (E)	LDL-c 2.6 mmol/L (N)
	HDL-c 1.7 mmol/L (N)	HDL-c 1.5 mmol/L (N)	HDL-c 1.3 mmol/L (N)
	TG 1.5 mmol/L (N)	TG 1.2 mmol/L (N)	TG 1.4 mmol/L (N)
Thyroid ultrasound examination	Enlarged, thickened thyroid gland, isoechogenic, fine granular structure, without nodules, with normal perfusion	Normal ultrasound report	Slightly enlarged, without nodules
Treatment needed	No	No	No

Legend: THRB, thyroid hormone receptor beta gene; TSH, thyrotropin; fT_4_, free thyroxin; fT_3_, free triiodothyronin; TC, total cholesterol levels; LDL-c, low-density lipoprotein cholesterol; HDL-c, high-density lipoprotein cholesterol; TG, triglycerides; N, normal; B, borderline; E, elevated.
